# An initial reliability analysis of a patient counseling rubric to objectively measure student pharmacist performance

**DOI:** 10.1016/j.heliyon.2023.e15768

**Published:** 2023-05-05

**Authors:** Kristin Ashley Garling, Benjamin Wong

**Affiliations:** a The University of Texas at Austin – College of Pharmacy, Texas, USA

**Keywords:** Patient counseling, Pharmacy student, Pharmacists, Reproducibility of results, Community pharmacy, Pharmacy education, Simulation training

## Abstract

Current literature outlines the documented need for improved communication during patient medication counseling. Although many tools exist, there needs to be a national standardized tool that complies with federal and state law, to objectively measure student pharmacist performance during patient counseling in the community pharmacy setting. The primary objective of this study is to perform an initial analysis of the internal consistency reliability of a patient medication counseling rubric designed with an Indian Health Services theoretical framework. Secondary objectives include measuring changes in student performance over the time of the study. The 18-item rubric was developed to objectively measure student pharmacist performance during patient medication counseling sessions in a 21-h Introductory Pharmacy Practice Experience (IPPE) course. The community-pharmacy-based IPPE patient counseling course evaluates students' communication skills and patient-centered counseling techniques in live and simulated patient counseling sessions. Three pharmacist evaluators assessed a total of 247 student counseling sessions. The rubric's internal consistency reliability was analyzed, and student performance improvement was observed within the course. Students' performance was evaluated as “meets expectations” in most live and simulated sessions. However, an independent groups *t*-test showed that the mean performance score for the live counseling sessions (2.59, SD = 0.29) was higher (p < 0.001) than that for the simulated counseling sessions (2.35, SD = 0.35). Students' performance in the course improved over three weeks [Week 1: mean (SD) = 2.29 (0.32), Week 2: mean (SD) = 2.44 (0.33), Week 3: mean (SD) = 2.62 (0.29); p < 0.001]. A Tukey-Kramer comparison post hoc test found a significant increase in the mean performance scores between weeks (p < 0.05). The overall internal consistency reliability of the counseling rubric was determined acceptable, with a Cronbach's alpha of 0.75. Further study is required, including the assessment of inter-rater reliability, factor analysis, variable analysis, and use in other states with patient confirmation testing necessary to validate the rubric for use with student pharmacists in the community pharmacy setting.

## Abbreviations

OBRA ‘90: Omnibus Budget Reconciliation Act of 1990
CAPE: Center for Advancement of Pharmacy Education
ACPE: Accreditation Council for Pharmacy Education
EPAs: Entrustable Professional Activities
TCEP: Texas Consortium on Experiential Programs
APhA: American Pharmacists Association
IPPE: Introductory Pharmacy Practice Experiences
P2: second-year student pharmacist
ANOVA: Analysis of variance
APPE: Advanced Pharmacy Practice Experiences
QuEST: Quickly and accurately assess the patient, Establish that the patient is an appropriate self-care candidate, Suggest appropriate self-care strategies, Talk with the patient
SCHOLARMAC: Symptoms, Characteristics, History, Onset, Location, Aggravating factors, Remitting factors, other Medications, Allergies, and Conditions

## 1 Introduction

Patient medication counseling (or patient counseling) is a discussion between a pharmacist and a patient to provide vital information to prevent medication-related problems such as inappropriate medication discontinuation, poor adherence, and prescription abandonment [1,2]. Nearly half of all prescribed medications are not taken by patients as prescribed, while 45.1% of patient populations across all medical specialties report medication-related problems [3,4]. Effective patient counseling can decrease the documented explanations for non-adherence, including cost, fear of adverse effects, polypharmacy, misunderstanding of the medication, condition, or expectations associated with treatment, and increase positive health outcomes [3,5]. Patient counseling mitigates high health risks while allowing patients to take an active, informed role in their healthcare [3,4].

Although patient counseling is critical to patient treatment, it must be more valued and practiced in pharmacy education [6,7]. In 1990, the Omnibus Budget Reconciliation Act of 1990 (OBRA’ 90) was signed into law to ensure patient counseling for Medicaid beneficiaries by pharmacists [8]. OBRA’ 90 aimed to improve patients' understanding of the medication their doctors prescribe [7,8]. Many states, including Texas, adopted similar administrative codes directing pharmacists to perform mandatory patient counseling or, at a minimum, offer counseling to all patients [9]. OBRA ‘90 and Texas law counseling requirements for patient medication counseling have minor differences (Appendix 1) [[8], [9], [10]]. In Texas, counseling is mandatory on new prescriptions or newly changed therapy components, such as dosing and frequency of the medication [9,10].

Despite being required by law, most counseling points are delivered in an ineffective passive lecture style [10]. Studies have found that only minimal medical information patients retain, with 40–80% being forgotten immediately [11]. It is also estimated that given the small percentage of medical information patients can retain, nearly half is misremembered [12,13]. Due to the high prevalence of medication-related problems and low accuracy of patient recall, it is clear that a more effective communication model is needed for student pharmacists [[3], [4], [5], [6], [7], [8], [9], [10]].

In 1991, the Indian Health Services created a theoretical framework to improve patient understanding and eliminate the traditional lecture style of patient counseling by utilizing three prime open-ended questions [14]. These open-ended questions are (1) “What did your doctor tell you about this medication?”, (2) “How did your doctor tell you to take the medication?” and (3) “What did your doctor tell you to expect?” These three prime questions aim to elicit patients' interpretations, feelings, and opinions about their medications [14,15]. The produced patient information, therefore, assists pharmacists in tailoring patient counseling to target any misunderstandings or gaps in the patient's medication knowledge and perception [2,6,15].

Although the Indian Health Services theoretical framework has been proven highly effective in studies, healthcare providers and student pharmacists have difficulty incorporating the method into practice [15]. Therefore, other concepts were developed, such as the QuEST (Quickly and accurately assess the patient, Establish that the patient is an appropriate self-care candidate, Suggest appropriate self-care strategies, Talk with the patient) process and the SCHOLAR-MAC (Symptoms, Characteristics, History, Onset, Location, Aggravating factors, Remitting factors, other Medications, Allergies, and Conditions) tool. Unfortunately, studies have shown that these additional concepts and techniques have similar obstacles to practice implementation or need to be more thorough for use as a standardized technique in all patient counseling situations [16,17].

Academic focus and educational variance are reported among the many barriers to pharmacy practice implementation [18]. In 2013, The Center for Advancement of Pharmacy Education (CAPE) Educational Outcomes and the Accreditation Council for Pharmacy Education (ACPE) incorporated requirements into the student pharmacist curricula to improve educational continuity [19,20]. A pivotal time to teach, implement, and practice these skills would be during a pharmacy student's community introductory pharmacy practice experiences (IPPE), typically held within the first three years of the Doctor of Pharmacy program [21,22]. The IPPE provides all students' required experiences and the initial and supervised opportunities for pharmacy students to practice didactic counseling techniques within practice sites with live patients [23].

Scoring rubrics, such as The Texas State Board of Pharmacy, designated the Texas Consortium on Experiential Programs (TCEP) Entrustable Professional Activities (EPAs), are needed to ensure sufficient competence of student pharmacist performance during an IPPE-related skill [24,25]. Although TCEP EPAs have been mandated for all IPPE student assessments since 2017, studies still need to be completed to analyze their use [26]. This study aims to measure the reliability of TCEP EPAs, in rubric form, utilized by pharmacist preceptors for student pharmacists during patient counseling sessions that follow the Indian Health Services theoretical framework. The primary objective of this study was to perform an initial internal consistency reliability analysis of a patient counseling rubric developed to objectively measure student pharmacist performance in an Introductory Pharmacy Practice Experience (IPPE) course. The secondary objective is to observe the changes in student performance over the time of the study.

## 2 Methodology

### 2.1 Rubric components and rubric scale development

Throughout 2018, the developing faculty member utilized student and teaching assistant feedback to improve the rubric items' use, order, and flow. The rubric was developed based on a literature review of best practices for pharmacist-led patient medication counseling in a community pharmacy setting [[14], [15], [16], [17]]. After identifying relevant literature, common themes were compared to the federal and state legal requirements for patient counseling [[7], [8], [9]]. Eighteen items consistent with legal requirements and consistently recognized as best practices were included as rubric items (Table 1).

**Table 1:** Performance distribution and mean performance score for each counseling rubric item in the live counseling sessions. (N = 170).

Medication counseling rubric item		Not applicable		Significant deficits exist		Needs improvement		Meets expectations		Exceeds expectations		Mean	(SD)
		N	(%)	N	(%)	N	(%)	N	(%)	N	(%)		
#1	What did the doctor tell you the medication was for?	0	(0.0)	3	(1.8)	0	(0.0)	167	(98.2)	0	(0.0)	2.96	(0.26)
#2	How did the doctor tell you to take the medication?	1	(0.6)	40	(23.5)	7	(4.1)	122	(71.8)	0	(0.0)	2.49	(0.85)
#3	What did the doctor tell you to expect?	0	(0.0)	63	(37.1)	9	(5.3)	98	(57.7)	0	(0.0)	2.21	(0.95)
#4	Identifies/introduces self as pharmacist	1	(0.6)	5	(2.9)	7	(4.1)	150	(88.2)	7	(4.1)	2.94	(0.45)
#5	Identifies/confirms patient or patient's agent	1	(0.6)	21	(12.4)	8	(4.7)	132	(77.7)	8	(4.7)	2.75	(0.73)
#6	Explains the purpose of the counseling session	25	(14.7)	29	(17.1)	17	(10.0)	90	(52.9)	9	(5.3)	2.54	(0.88)
#7	Provides medication name (Brand/Generic)	0	(0.0)	16	(9.4)	9	(5.3)	141	(82.9)	4	(2.4)	2.78	(0.64)
#8	Provides indication for medication (Use, preparation, expected action)	0	(0.0)	11	(6.5)	16	(9.4)	141	(82.9)	2	(1.2)	2.79	(0.57)
#9	Assesses patient understanding of the reason(s) for therapy and visually show the medication and/or device technique	0	(0.0)	18	(10.6)	11	(6.5)	135	(79.4)	6	(3.5)	2.76	(0.68)
#10	Provides dosage/regimen for medication (Route, Dosage Form, Dosage, Duration/Schedule)	0	(0.0)	3	(1.8)	12	(7.1)	152	(89.4)	3	(1.8)	2.91	(0.39)
#11	Use and update patient profile (address, phone, allergies, OTC/herbal medication, personal preferences)	1	(0.6)	69	(40.6)	9	(5.3)	87	(51.2)	4	(2.4)	2.15	(1.00)
#12	Discusses potential (major) side effects (Avoidance techniques, action if occurs, self-monitoring)	0	(0.0)	10	(5.9)	8	(4.7)	147	(86.5)	5	(2.9)	2.86	(0.54)
#13	Discusses potential warnings, precautions, and interactions (Therapeutic Contraindications)	7	(4.1)	23	(13.5)	25	(14.7)	115	(67.7)	0	(0.0)	2.56	(0.73)
#14	Describes missed dose instructions	27	(15.9)	41	(24.1)	10	(5.9)	91	(53.5)	1	(0.6)	2.36	(0.91)
#15	Provides number of refills or number of allowed refills	1	(0.6)	61	(35.9)	5	(2.9)	101	(59.4)	2	(1.2)	2.26	(0.97)
#16	Discusses storage recommendations (specify “room temperature”)	1	(0.6)	36	(21.2)	10	(5.9)	120	(70.6)	3	(1.8)	2.53	(0.85)
#17	Verifies patient understanding utilizing the Teach Back Method	0	(0.0)	32	(18.8)	13	(7.7)	121	(71.2)	4	(2.4)	2.57	(0.82)
#18	Summarizes by emphasizing key points of information, provides closure and opportunity for follow-up	0	(0.0)	65	(38.2)	20	(11.8)	84	(49.4)	1	(0.6)	2.12	(0.94)

The medication counseling rubric items included the non-validated American Pharmacist Association (APhA) annual counseling competition requirements, OBRA ‘90 federal requirements, and Texas state law requirements within an Indian Health Services theoretical framework [[8], [9], [10],^14^]. The rubric format starts with the students asking three foundational open-ended questions, the previously validated Indian Health Services Three Prime Questions [14]. The Three Prime Questions elicit responses that fulfill the OBRA ‘90 federal and Texas state law's patient counseling mandates [[8], [9], [10],^14^]. APhA best practices are recommended as further questions and techniques to be utilized within the patient encounter, ending with the validated teach-back method serving as a summary for the patient [12].

Throughout 2018, the developing faculty member utilized student and teaching assistant feedback to improve the use, order, and flow of the rubric items. Communications and community pharmacy preceptors performed semester-long pilot tests, in 2018–2019, with pharmacy students executing the rubric items. These pilots and student-provided feedback allowed for further refinement of item wording and rubric structure. Additionally, the pilots identified exclusion criteria and determined the weeks within the course that were most thorough for sampling.

### 2.2 IPPE course structure

The 18-item medication counseling rubric was utilized to evaluate students’ patient-centered counseling techniques in live and simulated patient counseling sessions. The Ethics and Institutional Review Board of the University of Texas in Austin approved this study with an exempt status in 2020. Furthermore, voluntary consent for the collection, analysis and publication of the retrospectively obtained and anonymized data for this non-interventional study was obtained by student participants.The rubric used for the study utilized an Indian Health Services theoretical framework for three weeks, mid-semester of a one-credit, 21-h Introductory Pharmacy Practice Experiences (IPPE) course. Students enrolled in the course in were attending their second year (P2) of the four-year Doctor of Pharmacy program.

**Fig. 1:**
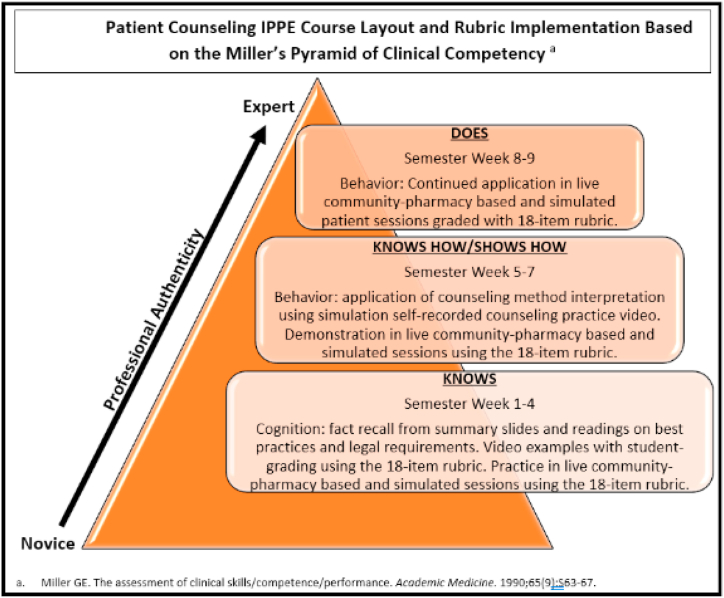
Miller's pyramid of clinical competence course progression.

The history, background, utilization, and purpose of these techniques and requirements of the course rubric were taught to students in a required didactic virtual classroom method. The virtual classroom utilized the online student platform, Canvas, to provide one location for students to access the compulsory course articles, slide presentations, and videos that supported the course rubric. These mandatory course components were due for completion by students before attending the in-class sessions according to the progression of Miller's Pyramid of Clinical Competence (Fig. 1.) [27]. In-class sessions were held in a community pharmacy for students to practice didactic techniques during pharmacist-led and evaluated simulation counseling and pharmacist-supervised live counseling with patients picking up new or newly transferred medications.

### 2.3 Implementation: study setting and sample

The location of the patient counseling occurred at Forty Acres Pharmacy, an independent university-based community pharmacy. The community pharmacy is not attributed to specialty medications or a hospital, allowing the public to receive both dispensed and compounded medications. Additionally, the community pharmacy enables the public to access additional medical services and medical information under the direct supervision of a pharmacist. The Forty Acres Pharmacy, located in the central campus area of the University of Texas at Austin, is a community pharmacy that serves over 74,000 students, faculty, staff, and people from the surrounding community. This study included face-to-face live patient counseling sessions with the primary end-user patient in a private counseling booth.

Live counseling sessions occurred in real-time and in person with patients receiving new and newly transferred prescriptions. Due to logistical issues and communication barriers, such as connectivity in virtual sessions, patient home distractors during telephonic sessions, and difficulty hearing patients’ responses during drive-thru sessions, these counseling sessions were excluded from the study.

Simulated counseling sessions were one-on-one sessions that occurred in real-time and in person with student pharmacists for practicing purposes. Simulated counseling sessions were utilized during slower volume times when patients were unavailable for live sessions. A faculty member or pharmacist graduate student acts as a patient with the patient's medical and social history, lifestyle preferences, and relevant therapy information provided. Uniform training occurred at the beginning of the semester for all three pharmacists to standardize the simulated patient counseling process and minimize reviewer bias. Simulated cases were developed based on Miller's Pyramid of Clinical Competence [27] and increased in complexity as the semester progressed.

### 2.4 Study design and data collection

The three pharmacist evaluators, one pharmacy faculty, and two doctors of pharmacy graduates self-reported EPA rating data over a nine-week semester course. The initial four weeks allowed graders to complete a 1-h training on using the rubric for student grading and practice grading student encounters, both live and simulated, to control variability and improve percent agreement between graders. Additionally, these initial four weeks of data were excluded from the analysis to account for the student's learning of the counseling method, outlined in the rubric, using Miller's Pyramid of Clinical Competence (Fig. 1) [27]. The learning steps in Miller's Pyramid of Clinical Competence [27] include knowledge gathering and cognition (KNOWS), which was attained by student pharmacists in the form of fact recall from summary slides and readings on the best practices and legal counseling requirements.

Additionally, student pharmacists’ subjects watched counseling videos and graded the simulation videos using the 18-item patient counseling rubric. The interpretation application steps (KNOWS HOW/SHOWS HOW) during weeks five through seven include student pharmacists recording themselves during a simulated counseling session to self-grade for improvement areas. During weeks eight and nine, the continued application (DOES) allowed additional practice in live and simulated counseling sessions for the student pharmacists. The data from weeks five through nine were thoroughly reviewed to exclude missing data, sessions requiring direct intervention from a licensed pharmacist, and data obtained from sessions with exclusion criteria. The remaining cleaned data included a three-week consecutive sample of total rating scores from the semester, and all sessions met all inclusion criteria.

### 2.5 Assessment

Each item on the 18-item medication counseling rubric carried equal weight and received a rating of 1–4 points from each evaluator based on the evaluator's perceptions of the student's performance. Four levels of rating categories were adapted from the Texas Consortium of Experiential Programs (TCEP) Entrustable Professional Activity (EPA) Statements (Appendix 2). The TCEP's and EPA's methods for scale development were not available for inclusion in this study.

For the TCEP rating scale, a rating of 1 indicates “significant deficits exist when the student has rarely demonstrated the competency at an acceptable level and often does not complete tasks requiring continual guidance from the preceptor.” The EPA level of entrustment in this rating category is – “I trust the learner to perform the task only with proactive supervision and/or frequent correction.” A TCEP rating of 2 indicates “needs improvement when the student has not consistently demonstrated the competency at an acceptable level and requires frequent guidance from the preceptor.” The EPA level of entrustment in this rating category is – “I trust the learner to perform the task with supervision and/or correction.” A rating of 3 indicates “meets expectations when the student has performed the competency at an acceptable level and met preceptor expectations but requires occasional guidance.” The level of entrustment in this rating category is – I trust the learner to perform the task with limited supervision and/or correction.” A rate of 4 indicates “exceeds expectations when the student has excelled in performing competency and performs above expectations requiring minimal guidance from the preceptor.” The level of entrustment in this rating category is – “I trust the learner to perform the assigned task.”

### 2.6 Data analysis

All statistical analyses were conducted using SAS software, version 9.4 (SAS Institute Inc. Cary, NC), with two-sided statistical tests at a 0.05 significance level. Descriptive statistics were calculated for student performance scores in live and simulated counseling sessions, with the means and standard deviations being reported. Independent groups t-tests assessed the relationship between the student performance scores for live and simulated counseling sessions. A one-way analysis of variance (ANOVA) model compared the student performance scores across the three-week sessions. Cronbach's alpha was used to assess the internal consistency reliability of the counseling rubric.

## 3 Results

There were 247 student counseling sessions (77 simulated and 170 live), meeting inclusion criteria, evaluated by pharmacy faculty and graduate student pharmacists. The overall internal consistency reliability of the counseling rubric, Cronbach's alpha, was calculated to be 0.75.

Table 1 summarizes the performance distribution and the mean performance score for each counseling rubric item in the live counseling sessions. The pharmacist evaluators evaluated students as “meets expectations” in most live counseling sessions. Almost all students (N = 167, 98.2%) met expectations in Item 1, “What did the doctor tell you the medication was for?” and had the highest mean score (mean = 2.96, SD = 0.26), followed by Item 4 “Identifies/introduces self as a pharmacist” where students had a mean score of 2.94 (SD = 0.45). Students had the lowest mean score in Item 18, “Summarize by emphasizing key points of information, provides closure and opportunity for follow-up” (mean = 2.12, SD = 0.94), where 65 (38.2%) and 84 (49.4%) students were evaluated as “significant deficits exit” and “meets expectations,” respectively.

Table 2 summarizes the performance distribution and the mean performance score for each counseling rubric item in the simulated counseling sessions. Students' performance varied in the simulated counseling sessions; however, most students were evaluated as “meets expectations” by their pharmacist evaluators. Many students (N = 66, 85.7%) met expectations in Item 4, “Identifies/introduces self as a pharmacist,” and had the highest mean score (mean = 2.82, SD = 0.56), followed by Item 1, “What did the doctor tell you the medication was for?” where students had a mean score of 2.68 (SD = 0.52). Students had the lowest mean score in Item 11, “Use and update patient profile (address, phone, allergies, OTC/herbal medication, personal preferences)” (mean = 1.97, SD = 0.84), where 28 (36.4%) and 26 (33.7%) students were evaluated as “significant deficits exit” and “meets expectations,” respectively.

**Table 2:** Performance distribution and mean performance score for each counseling rubric item in the simulated counseling sessions (N = 77).

Medication counseling rubric item		Significant deficits exist (Rating 1)		Needs improvement (Rating 2)		Meets expectations (Rating 3)		Exceeds expectations (Rating 4)		Mean	(SD)
		N	(%)	N	(%)	N	(%)	N	(%)		
#1	What did the doctor tell you the medication was for?	2	(2.6)	21	(27.3)	54	(70.1)	0	(0.0)	2.68	(0.52)
#2	How did the doctor tell you to take the medication?	10	(13.0)	31	(40.3)	36	(46.8)	0	(0.0)	2.34	(0.70)
#3	What did the doctor tell you to expect?	16	(20.8)	23	(29.9)	38	(49.4)	0	(0.0)	2.29	(0.79)
#4	Identifies/introduces self as pharmacist	5	(6.5)	5	(6.5)	66	(85.7)	1	(1.3)	2.82	(0.56)
#5	Identifies/confirms patient or patient's agent	10	(13.0)	19	(24.7)	48	(62.3)	0	(0.0)	2.49	(0.72)
#6	Explains the purpose of the counseling session	23	(29.9)	29	(37.7)	24	(31.2)	1	(1.3)	2.04	(0.82)
#7	Provides medication name (Brand/Generic)	4	(5.2)	33	(42.9)	40	(52.0)	0	(0.0)	2.47	(0.60)
#8	Provides indication for medication (Use, preparation, expected action)	9	(11.7)	29	(37.7)	39	(50.7)	0	(0.0)	2.39	(0.69)
#9	Assesses patient understanding of the reason(s) for therapy and visually show the medication and/or device technique	15	(19.5)	19	(24.7)	43	(55.9)	0	(0.0)	2.36	(0.79)
#10	Provides dosage/regimen for medication (Route, Dosage Form, Dosage, Duration/Schedule)	4	(5.2)	28	(36.4)	45	(58.4)	0	(0.0)	2.53	(0.60)
#11	Use and update patient profile (address, phone, allergies, OTC/herbal medication, personal preferences)	28	(36.4)	23	(29.9)	26	(33.8)	0	(0.0)	1.97	(0.84)
#12	Discusses potential (major) side effects (Avoidance techniques, action if occurs, self-monitoring)	7	(9.1)	25	(32.5)	44	(57.1)	1	(1.3)	2.51	(0.68)
#13	Discusses potential warnings, precautions, and interactions (Therapeutic Contraindications)	17	(22.1)	35	(45.5)	25	(32.5)	0	(0.0)	2.10	(0.74)
#14	Describes missed dose instructions	27	(35.1)	19	(24.7)	31	(40.3)	0	(0.0)	2.05	(0.87)
#15	Provides number of refills or number of allowed refills	20	(26.0)	13	(16.9)	43	(55.8)	1	(1.3)	2.32	(0.88)
#16	Discusses storage recommendations (specify “room temperature”)	20	(26.0)	18	(23.4)	36	(46.8)	3	(3.9)	2.29	(0.90)
#17	Verifies patient understanding utilizing the Teach Back Method	17	(22.1)	21	(27.3)	38	(49.4)	1	(1.3)	2.30	(0.83)
#18	Summarizes by emphasizing key points of information, provides closure and opportunity for follow-up	5	(6.5)	37	(48.1)	35	(45.5)	0	(0.0)	2.39	(0.61)

Table 3 summarizes the mean performance scores for the live and simulated counseling sessions. An independent groups *t*-test showed the mean performance score for the live counseling sessions (2.59, SD = 0.29) was significantly higher than that for the simulated counseling sessions (2.35, SD = 0.35) (t = −5.15; df = 125.21; p < 0.001). Students' performance in the course improved over three weeks, serving as data for further tool validation. Table 4 summarizes the mean performance scores for all applicable counseling sessions (N includes live and simulated sessions) over three weeks. A one-way ANOVA showed a difference in the mean performance scores over three weeks (F = 19.65; df = 2, 244; p < 0.001). A Tukey-Kramer comparison post hoc test found a significant increase in the mean performance scores between Week 3 and Week 2, Week 3 and Week 1, and Week 2 and Week 1, at an alpha level of p < 0.05.

**Table 3:** Mean performance scores for the live and simulated counseling sessions.

Counseling sessions	N	Mean	(SD)	p-value
Live	170	2.59	(0.29)	< **0.001**
Simulated	77	2.35	(0.35)

**Table 4:** Mean performance scores of all counseling sessions over the course of three weeks.

Time	N	Mean	(SD)	p-value
Week 1	34	2.29^b^^,^^c^	0.32	<0.001^d^
Week 2	84	2.44^a^^,^^c^	0.33	
Week 3	129	2.62^a^^,^^b^	0.29	

a Significant Tukey-Kramer comparison, defined as p-value< 0.05, for Week 3 vs. Week 2.

b Significant Tukey-Kramer comparison, defined as p-value< 0.05, for Week 3 vs. Week 1.

c Significant Tukey-Kramer comparison, defined as p-value< 0.05, for Week 2 vs. Week 1.

d Overall comparison.

## 4 Discussion

Current literature outlines the documented need for improved communication among pharmacists during patient counseling [[1], [2], [3], [4], [5], [6], [7], [8], [9], [10], [11], [12]]. Although many tools for patient counseling exist, neither a national standardized instrument nor validated best practices to objectively measure student performance during patient counseling is available [[14], [15], [16], [17]]. The primary objective of this study was to perform an initial internal consistency reliability analysis of a newly developed patient counseling rubric created to objectively measure student pharmacist performance in an Introductory Pharmacy Practice Experience (IPPE) course. With further testing of inter-rater reliability, factor analysis, variable analysis, and use in other states with patient confirmation testing, the researcher intends that this rubric will become a validated tool for student-pharmacist evaluation in pharmacy curricula nationwide.

Existing literature, Seybert et al. [28], Vyas et al. [29], and Seybert et al. [30] currently describe pharmacy students' performance in patient counseling simulations; however, these studies focused on patient simulations in critical and acute care settings, not community settings [[28], [29], [30], [31]]. While our analysis focused on community pharmacy IPPE scenarios, we found a similar trend as the supporting studies in improving students' performance scores in medication counseling sessions. No other studies that included live and simulated patient counseling sessions in a community setting were available for comparison.

Our study also found that the patient medication counseling rubric based on the standards set by the Texas Consortium on Experiential Programs Entrustable Professional statements had acceptable internal consistency reliability with a Cronbach's alpha value of 0.75. This value indicates that the rubric can be adopted to assess pharmacy student performance in medication counseling in the community pharmacy setting after further testing and validation.

Our study's overall mean performance score for the live counseling sessions was statistically significantly higher than that for the simulated sessions. The lower overall mean score for the simulated counseling sessions was attributable to a higher percentage of students rated as “needs improvements” than the live counseling sessions. The difference between live and simulated sessions was attributed to the need for more impromptu responses from the students, which increased the graders' leniency. For example, if a patient stated a side effect in addition to administration, “I take it twice a day with food to avoid nausea,” this would negate the next question, Item 3, “What did the doctor tell you to expect?”. When additional information was presented that negates further questions, a student pharmacist was either rated as “not applicable (N/A)” or graded as “meets expectations” or “exceeds expectations” if they were able to augment the question effectively.

Among the counseling rubric items, Item 1, “What did the doctor tell you the medication was for?” and Item 4, “Identifies/introduces self as a pharmacist,” were the top two items where students had the highest performance scores in both simulated and live counseling sessions, whereas items with the lowest performance scores varied between simulated and live counseling sessions. We found that students had the lowest performance scores in Item 11 and Item 18. Item 11, “Use and update patient profile,” requires access to the patient's electronic record and includes additional training for dispensing technology utilization outside the IPPE course scope. The component of Item 18, “Summarize by emphasizing key points of information, provides closure and opportunity for follow-up,” immediately follows Item 17 teach-back, which also provides the necessary summary of the patient counseling session, thus making Item 18 redundant.

When analyzing student performance, the authors used the number of sessions as the single data point for the one-way ANOVA. The difference in the number of sessions was due to patient counseling availabilities within the pharmacy setting and the removal of sessions that did not fit the inclusion criteria. The authors also recognize that with the current study design, each student contributed scores from multiple sessions to the data. This possibility of having correlated data may lead to a type 1 error that requires further study. Furthermore, our study did not include individual item factors and variable assessments that would have strengthened our findings.

Limitations in sample size, length of study, study design, and the need for further reliability analysis must be considered when interpreting the results of this study. First, our study was limited to a single P2 class at a single college of pharmacy in one state and conducted within a university-based community pharmacy yielding a limited sample size. Secondly, our study had a short evaluation period (up to three weeks). A longer follow-up evaluation period would allow students to refine and improve their medication counseling skills during their IPPE. Thus, the results need to be more generalizable to future class cohorts, other colleges, other states, or other clinical settings (e.g., critical care or ambulatory care pharmacy).

Future studies should consist of the study of the rubric with independent/dependent variable regression analysis, a more extended follow-up evaluation period, inter-rater reliability, predictive validity, confirmatory factor analysis, patient confirmation assessment, and study of the rubric in other classes/colleges/community pharmacies/states, and exploring how the usability translates across varying legal requirements between states are additional tests to create a verified rubric.

## 5 Conclusion

Pharmacists’ effective communication and counseling skills are imperative for improving the use of medications by patients, leading to optimal treatment outcomes [3,[5], [32]]. Most organizations, such as the Accreditation Council for Pharmacy Education, have stressed these essential skill sets [19,[20], [33]] and encouraged the incorporation of a standardized rubric to bridge the gap between didactic pharmacy education and effective live patient counseling [[34], [35], [36]]. Our 18-item rubric for effective student pharmacist counseling in the community pharmacy setting incorporates the Indian Health Services, the federal and state patient counseling legal requirements, and additional professional communication best practices; it was successfully analyzed for internal consistency reliability. Additionally, student performance scores improved throughout the IPPE-based community pharmacy course. These findings can serve as initial evidence for using the 18-item rubric. Further study is required to determine inter-rater reliability, factor analysis, variable analysis, and use in other states with patient confirmation testing to validate the rubric for use with student pharmacists. Future research goals are additional development and analysis to provide a standardized, proficient, reliable, and validated patient counseling rubric for use in simulated and live patient community pharmacy encounters for pharmacy colleges.

## Author contribution statement

Ashley Garling - conceived and designed the experiments; performed the experiments; analyzed and interpreted the data; contributed reagents, materials, analysis tools or data; wrote the paper. Benjamin Wong - conceived and designed the experiments; performed the experiments; analyzed and interpreted the data; contributed reagents, materials, analysis tools or data; wrote the paper.

## Funding information

The authors received no financial support for the research, authorship, and/or publication of this article.

## Data availability statement

Data included in article/supp. material/referenced in article.

## Declaration of interest's statement

The authors declare no competing interests.

**Table:** 

Appendix 1. OBRA 90′ Federal Patient Counseling Law versus Texas State Law [[8], [9], [10]]	
**OBRA 90′ Federal**	**Texas State**
Name of Drug (brand, generic, or other description)	Name and description of the drug or device*
Intended use and expected action*	
Route of administration	Route of administration
Dosage form	Dosage form
Dosing instructions	Dosing instructions
Administration schedule*	Duration of drug therapy*
Self-monitoring techniques	Self-monitoring techniques
	Special directions and precautions for:
	• Preparation
	• Administration
	• Use by the patient*
Common side effects	Common severe side/adverse effects
• Avoidance technique	• Avoidance technique
• The action if they occur	• The action if they occur
Proper storage	Proper storage
Potential drug-drug, drug-food, and other contraindications	Interactions and therapeutic contraindications*
Refill information	Refill information
Action if missed a dose	Action if missed
*Differences indicated between federal and state law	

## References

[1] Tariq R.A., Vashisht R., Sinha A., Scherbak Y. (2022). StatPearls.

[2] Akinbosoye O.E., Taitel M.S., Grana J., Hill J., Wade R.L. (2016). Improving medication adherence and health care outcomes in a commercial population through a community pharmacy. Popul. Health Manag..

[3] Volino L.R., Das R.P., Mansukhani R.P., Cosler L.E. (2014). Evaluating the potential impact of pharmacist counseling on medication adherence using a simulation activity. Am. J. Pharmaceut. Educ..

[4] Garin N., Sole N., Lucas B. (2021). Drug related problems in clinical practice: a cross-sectional study on their prevalence, risk factors and associated pharmaceutical interventions. Sci. Rep..

[5] Brown M.T., Bussell J.K. (2011). Medication adherence: WHO cares?. Mayo Clin. Proc..

[6] Ilardo M.L., Speciale A. (2020). The community pharmacist: perceived barriers and patient-centered care communication. Int. J. Environ. Res. Publ. Health.

[7] Showande S.J., Laniyan M.W. (2021). Patient medication counselling in community pharmacy: evaluation of the quality and content. J. Pharm. Policy Pract..

[8] Text - H.R.5835 - 101st Congress (1989-1990): Omnibus Budget Reconciliation Act of 1990, 1990, November 5, https://www.congress.gov/bill/101st-congress/house-bill/5835/text.

[9] Texas Administrative Code, 2007. Minimum requirements for Treatment of Chronic Pain. https://texreg.sos.state.tx.us/public/readtac$ext.

[10] (1997). ASHP guidelines on pharmacist-conducted patient education and counseling. Am. J. Health Syst. Pharm..

[11] Kessels R.P. (2003). Patients' memory for medical information. J. R. Soc. Med..

[12] Masters K. (2013). Edgar Dale's Pyramid of Learning in medical education: a literature review. Med. Teach..

[13] Brega A., Barnard J., Mabachi N. (2015). https://www.ahrq.gov/health-literacy/improve/precautions/toolkit.html.

[14] Colvin N.N., Mospan C.M., Buxton J.A., Waggett J.D., Gillette C. (2018). Using Indian Health Service (IHS) counseling techniques in an independent community pharmacy to improve adherence rates among patients with diabetes, hypertension, or hyperlipidemia. J. Am. Pharm. Assoc. JAPhA.

[15] Lam N., Muravez S.N., Boyce R.W. (2015). A comparison of the Indian Health Service counseling technique with traditional, lecture-style counseling. J. Am. Pharm. Assoc. JAPhA.

[16] Leibowitz K., Ginsburg D. (2002).

[17] Buring S.M., Kirby J., Conrad W.F. (2007). A structured approach for teaching students to counsel self-care patients. Am. J. Pharmaceut. Educ..

[18] McDonough R.P., Rovers J.P., Currie J.D., Hagel H., Vallandinghanl J., Sobotka J. (1998). Obstacles to the implementation of pharmaceutical care in the community setting. J. Am. Pharmaceut. Assoc..

[19] Medina M.S., Plaza C.M., Stowe C.D., Robinson E.T., DeLander G., Beck D.E., Melchert R.B., Supernaw R.B., Roche V.F., Gleason B.L., Strong M.N., Bain A., Meyer G.E., Dong B.J., Rochon J., Johnston P. (2013). Center for the advancement of pharmacy education 2013 educational outcomes. Am. J. Pharmaceut. Educ..

[20] Vlasses P.H., Wadelin J.W., Travlos D.V. (2008). Accreditation council for pharmacy education: annual report. Am. J. Pharmaceut. Educ..

[21] Medina M.S., Plaza C.M., Stowe C.D., Robinson E.T., DeLander G., Beck D.E., Melchert R.B., Supernaw R.B., Roche V.F., Gleason B.L., Strong M.N., Bain A., Meyer G.E., Dong B.J., Rochon J., Johnston P. (2013). Center for the advancement of pharmacy education 2013 educational outcomes. Am. J. Pharmaceut. Educ..

[22] Teeter B., Stafford R., Payakachat N., Reid J., Thiessen K., Franks A. (2019). Student pharmacists' use of patient-centered communication skills during an introductory pharmacy practice experience. Am. J. Pharmaceut. Educ..

[23] Rathbone A.P., Richardson C.L., Mundell A., Lau W.M., Nazar H. (2021). Exploring the role of pharmacy students using entrustable professional activities to complete medication histories and deliver patient counselling services in secondary care. Explor. Res. Clin. Soc. Pharm..

[24] Haines S.T., Pittenger A.L., Stolte S.K., Plaza C.M., Gleason B.L., Kantorovich A., McCollum M., Trujillo J.M., Copeland D.A., Lacroix M.M., Masuda Q.N., Mbi P., Medina M.S., Miller S.M. (2017). Core entrustable professional activities for new pharmacy graduates. Am. J. Pharmaceut. Educ..

[25] Preceptor Requirements and Ratio of Preceptors to Pharmacist-Interns, 2019, Texas administrative code. https://texreg.sos.state.tx.us/public/readtac$ext.

[26] LexisNexis Editorial Staff (2021).

[27] Miller G.E. (1990). The assessment of clinical skills/competence/performance. Acad. Med..

[28] Ling Y., Leng L.W. (2013). Pharmacy student response to patient simulation based learning. Procedia Soc. Behav. Sci..

[29] Vyas D., Wombwell E., Russell E., Caligiuri F. (2010). High-fidelity patient simulation series to supplement introductory pharmacy practice experiences. Am. J. Pharmaceut. Educ..

[30] Seybert A.L., Kane-Gill S.L. (2011). Elective course in acute care using online learning and patient simulation. Am. J. Pharmaceut. Educ..

[31] Baumgartner L., Ip E.J., Sasaki-Hill D., Wong T., Israel H., Barnett M.J. (2019). Implementation of mock acute care advance pharmacy practice experience simulations and an assessment rubric. Am. J. Pharmaceut. Educ..

[32] Boissy A., Windover A.K., Bokar D., Karafa M., Neuendorf K., Frankel R.M., Merlino J., Rothberg M.B. (2016). Communication skills training for physicians improves patient satisfaction. J. Gen. Intern. Med..

[33] Coulter A., Ellins J. (2007). Effectiveness of strategies for informing, educating, and involving patients. BMJ.

[34] Ford C.R., Garza K., Kavookjian J., Kleppinger E.L. (2019). Assessing student pharmacist communication skills: development and implementation of a communication rubric. Curr. Pharm. Teach. Learn..

[35] Wallman A., Vaudan C., Sporrong S.K. (2013). Communications training in pharmacy education, 1995–2010. Am. J. Pharmaceut. Educ..

[36] Marken P.A., Zimmerman C., Kennedy C., Schremmer R., Smith K.V. (2010). Human simulators and standardized patients to teach difficult conversations to interprofessional health care teams. Am. J. Pharmaceut. Educ..

